# Supplementary material for: Budding yeast Rif1 binds to replication origins and protects DNA at blocked replication forks
Source: EMBO Rep. 2018 Aug 13;19(9):e46222. doi: 10.15252/embr.201846222 (PMC6123642; doi:10.15252/embr.201846222)
Supplement: Supplementary file 1 — Expanded View Figures PDF [file EMBR-19-e46222-s001.pdf]

Expanded View Figures

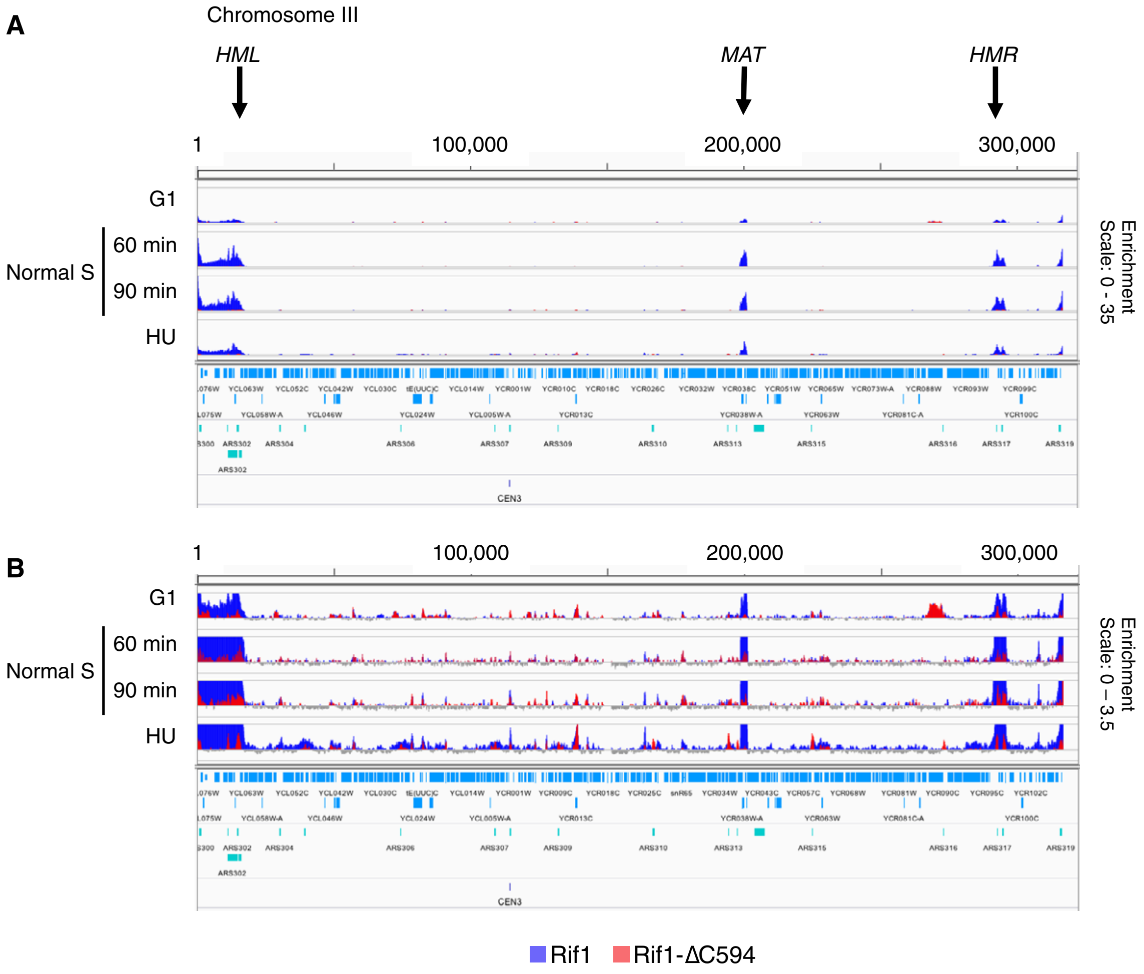

Figure EV1. Rap1-dependent association of Rif1, but not Rif1-ΔC, with telomeres, *MAT* locus and mating type cassettes.

A Enrichment of ChIP signal relative to Input DNA of Rif1 (blue) and Rif1-ΔC594 (red) along entire chromosome III. *Y*-axis scale is 0–35.

B Same data with *Y*-axis scale 0–3.5.

© 2018 The Authors

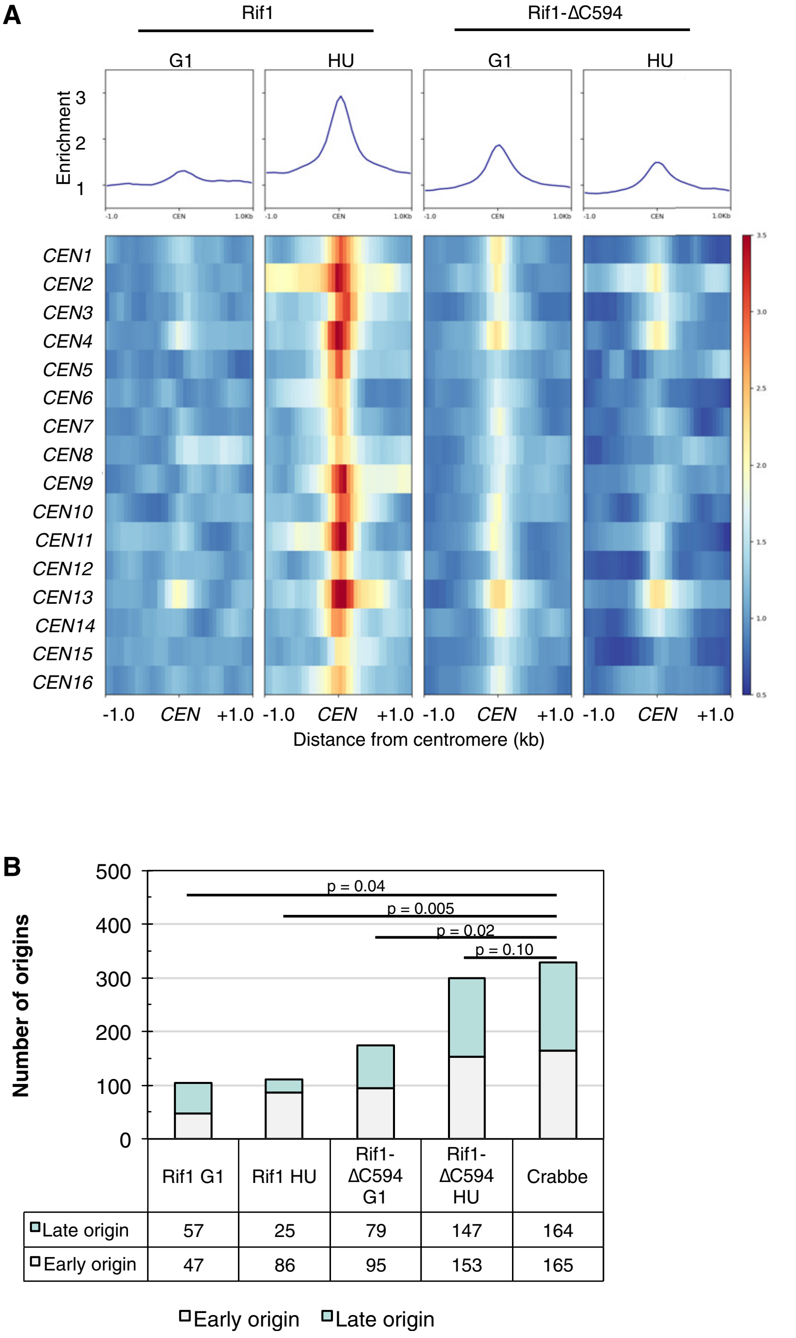

Figure EV2. Association of Rif1 and Rif1-ΔC594 with centromeres and DNA replication origins.

A Association of Rif1 protein with centromeres. *Top*. Average ChIP profiles at all 16 centromeres of Rif1 and Rif1-ΔC594 in G1 phase and HU-arrested cells. *Bottom*. Heat maps showing Rif1 and Rif1-ΔC594 ChIP enrichment at individual centromeres.

B Association of Rif1 and Rif1-ΔC594 with replication origins. Locations of ChIP peaks, identified by peak-calling algorithm MACS2, were compared with locations of origins of known replication timing (shown as “Crabbe”) [34]. Subtelomeric origins (< 15 kb from chromosome ends) were excluded from this analysis. Distributions of peaks detected at early and late origins were statistically tested against distribution of total origins with known replication timing, using hypergeometric distribution. The *P*-values obtained are shown above.

© 2018 The Authors

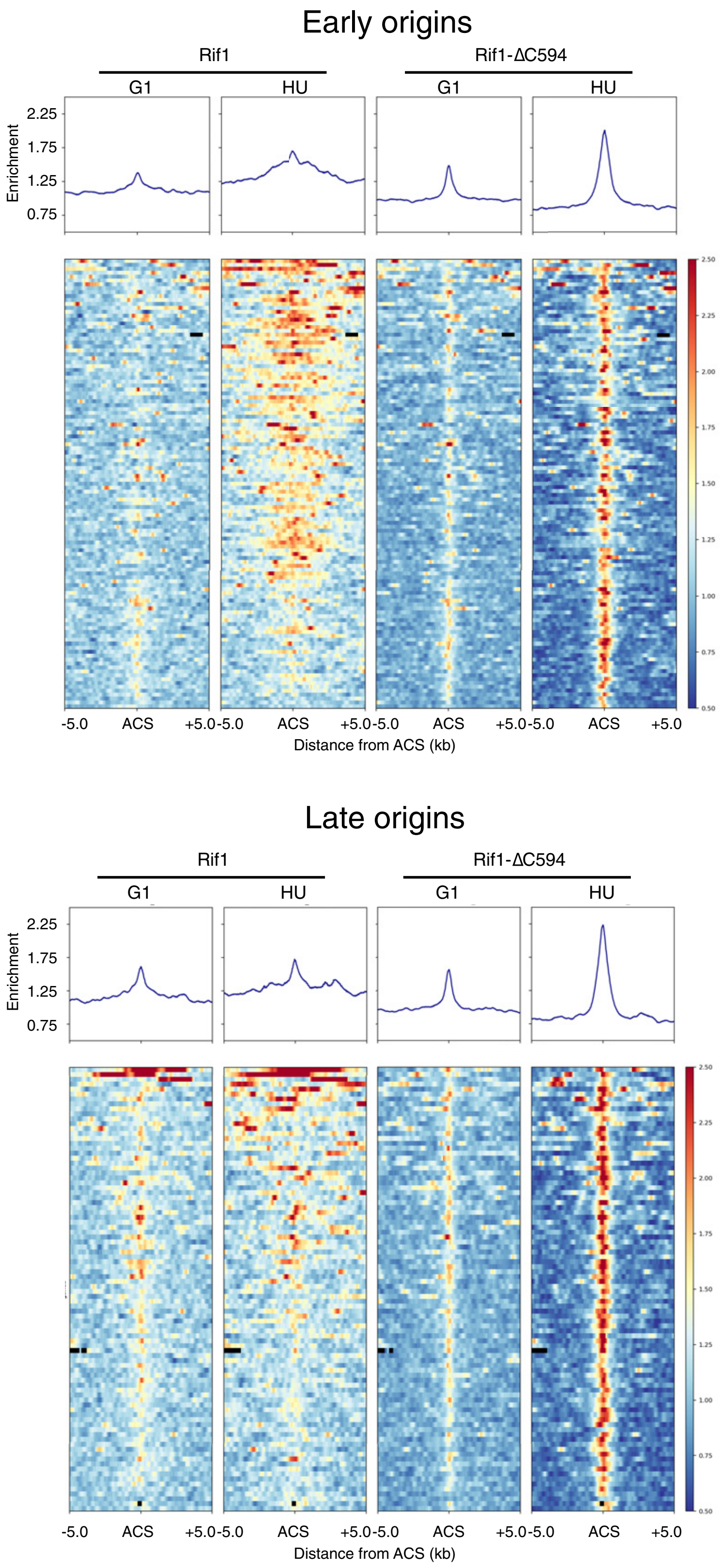

Figure EV3. Average and heat map presentation of Rif1 and Rif1-ΔC594 ChIP profiles at replication origins.

Rif1 and Rif1-ΔC594 ChIP signals were aligned for the all DNA replication origins with known replication timing and predicted ARS Consensus Sequence (ACS) [34,63]. Origins are centred on the predicted ACS site. Top panels show the average ChIP profiles and heat maps at the 115 early initiating origins, and the bottom panel shows those at the 90 late-initiating origins. The two late origin sites showing particularly strong, broad Rif1 ChIP signals (appearing at top of "Late origins Rif1" panels) correspond to the replication origins located within mating type cassettes.

© 2018 The Authors

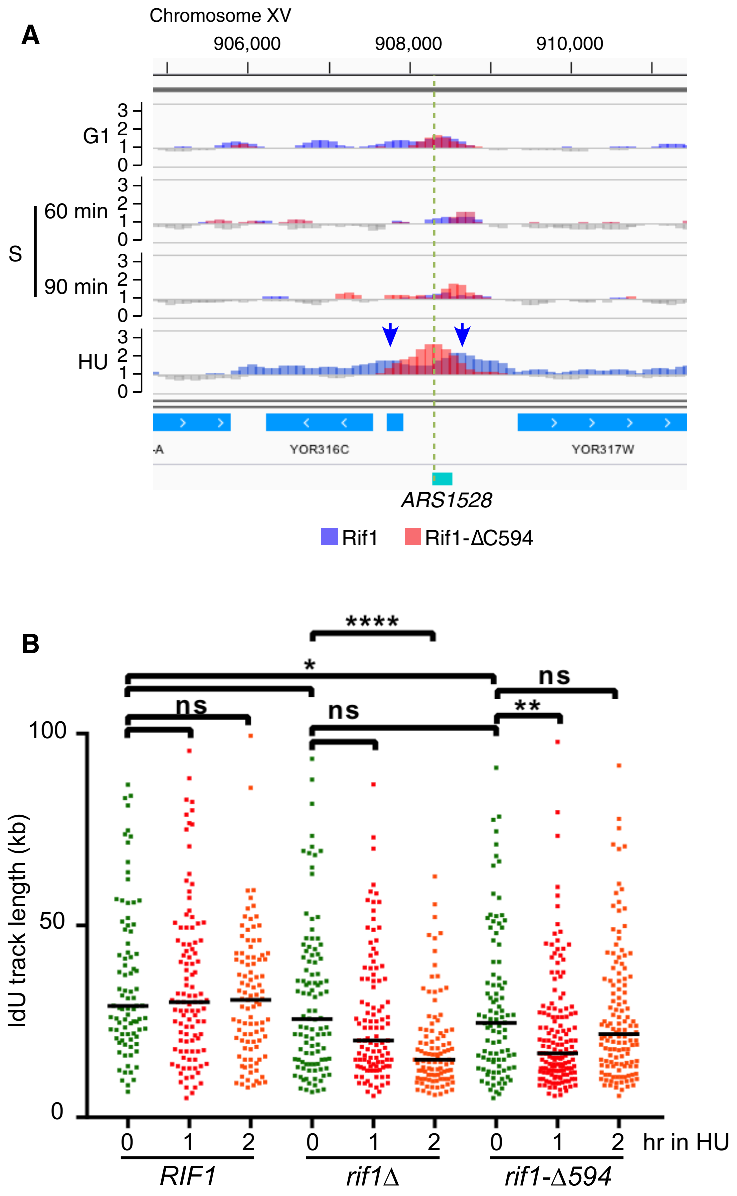

Figure EV4. Association of Rif1 with replication forks and protection of nascent DNA.

A Split Rif1 ChIP peak observed at *ARS1528* in HU. ChIP profiles of Rif1 and Rif1-ΔC594 are shown as in Fig 2B. Splitting of Rif1 peak indicated by blue arrows. Position of the predicted ACS for *ARS1528* marked by a green dotted line.

B Rif1 protects nascent DNA from degradation at stalled forks. Protection of nascent DNA from degradation was assayed as in Fig 5, but with slightly longer labelling time (22 min). Cells were blocked in HU for 0, 1 and 2 h after IdU labelling. Numbers above the dot plots indicate the number of tracts examined. Results were compared using Mann–Whitney–Wilcoxon test. ns, not significant. *P-value < 0.05, **P-value < 0.01, ****P-value below 0.0001.

Figure EV5. Association of Rif1 and Rif1-ΔC594 with genes encoding ribosomal protein.

A ChIP-Seq profiles of Rif1 and Rif1-ΔC594 at the *RPS31* gene. ChIP enrichment of Rif1 and Rif1-ΔC594 at the *RPS31* gene, whose transcription is controlled by Rap1 [32], presented as in Fig 2B.

B Rif1 and Rif1-ΔC594 binds ribosomal protein genes. Average and heat map presentations of Rif1 and Rif1-ΔC594 binding at 136 ribosomal protein-encoding genes [64]; 56 are genes for small ribosomal proteins (*RPS* genes) and 80 are genes for large ribosomal proteins (76 *RPL* and 4 *RPP* genes). Genes for two ribosome-like proteins (*RLP7* and *RLP24*) are excluded from the analysis. Both Rif1 and Rif1-ΔC594 show enrichment near the stop codon. Full-length Rif1 also shows association within coding sequences, largely absent for Rif1-ΔC594. Rif1, but not Rif1-ΔC594, also shows binding to promoter regions, as expected since many ribosome-encoding genes are regulated by Rap1 [40].

© 2018 The Authors

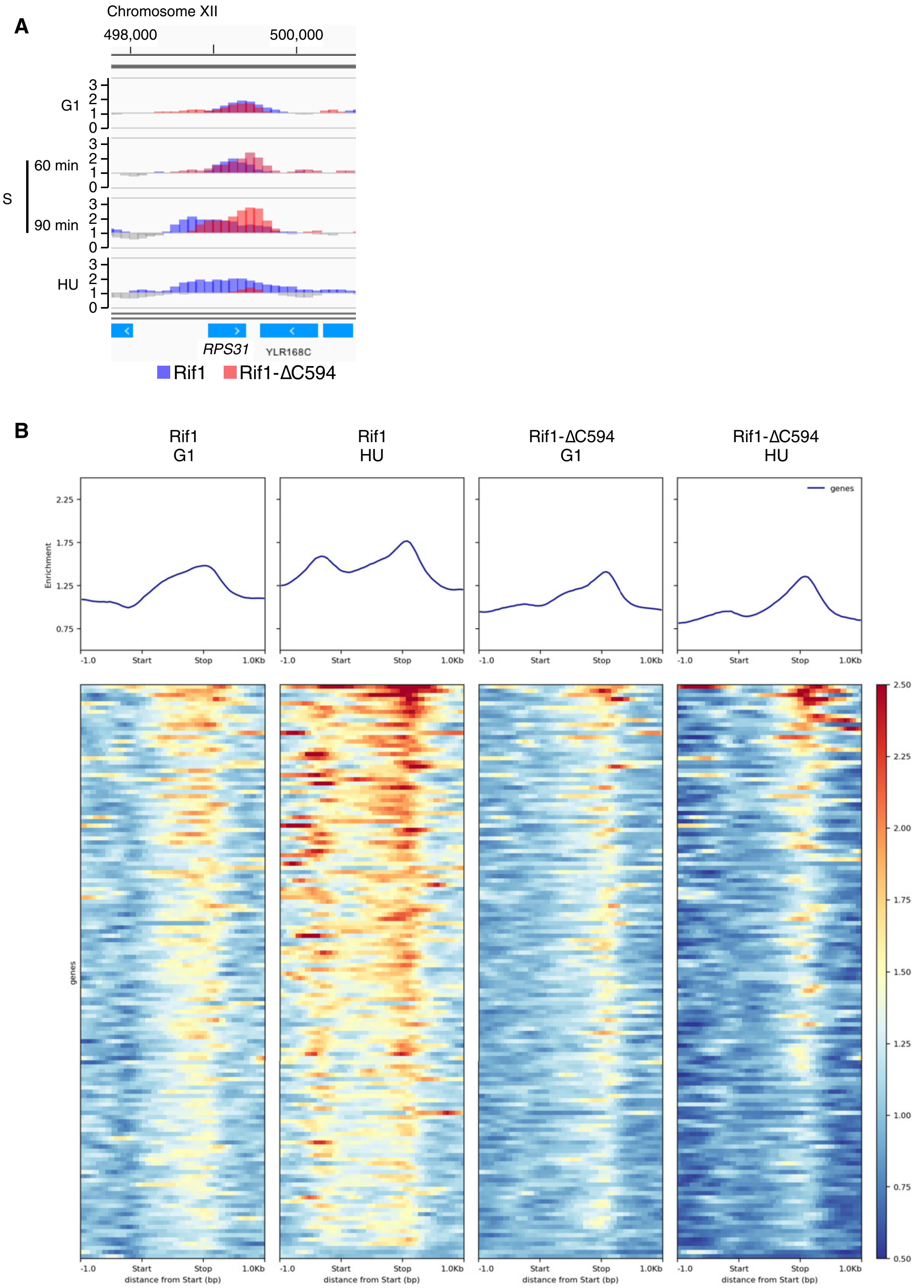
A
Chromosome XII
498,000
500,000
G1
S
60 min
90 min
HU
RPS31
YLR168C
Rif1
Rif1-ΔC594
B
Rif1
G1
Rif1
HU
Rif1-ΔC594
G1
Rif1-ΔC594
HU
genes
Enrichment
Start
Stop
1.0Kb
genes
distance from Start (bp)

Figure EV5.
