# Supplementary material for: Budding yeast Rif1 binds to replication origins and protects DNA at blocked replication forks
Source: EMBO Rep. 2018 Aug 13;19(9):e46222. doi: 10.15252/embr.201846222 (PMC6123642; doi:10.15252/embr.201846222)
Supplement: Supplementary file 2 — Table EV1 [file EMBR-19-e46222-s002.docx]

**Table EV1. Statistical evaluation of Rif1 association with genes with intron**

| Rif1 or Rif1-∆C594 occupancy | Number of genes | Genes with intron | P-value |
| --- | --- | --- | --- |
| >90% | 194 | 16 | 0.018 |
| >85% | 220 | 20 | 4.08 x 10^-3^ |
| >80% | 245 | 23 | 1.59 x 10^-3^ |
|  |  |  |  |
| All genes | 5,156 | 262 |  |

Genes showing occupancy by Rif1 or Rif1-∆C594 extending the indicated percentage of their coding sequence (under any condition) were selected. Within each group the enrichment of genes containing an intron was then tested statistically, using hypergeometric distribution.
