# Supplementary material for: Budding yeast Rif1 binds to replication origins and protects DNA at blocked replication forks
Source: EMBO Rep. 2018 Aug 13;19(9):e46222. doi: 10.15252/embr.201846222 (PMC6123642; doi:10.15252/embr.201846222)
Supplement: Supplementary file 3 — Table EV2 [file EMBR-19-e46222-s003.docx]

**Table EV2. Comparison of Rif1 ChIP peaks with S1-DRIP sites and predicted G4 sites**

|  | | Rif1 ChIP | | | | |
| --- | --- | --- | --- | --- | --- | --- |
|  |  | Rif1 | | Rif1-∆C594 | | Randomised^1^ |
|  |  | G1 | HU | G1 | HU |  |
| Number of ChIP peaks | | 762 | 915 | 883 | 1592 | 1592 |
|  | |  |  |  |  |  |
| Overlap with;^2^ | S1-DRIP-Seq (total 779) | 164 | 208 | 199 | 252 | 67 |
|  |  |  |  |  |  |  |
|  | G4 including subtelomeric  (total 636) | 98 | 140 | 45 | 149 | 57 |
|  | G4 excluding subtelomeric (total 544) | 26 | 62 | 28 | 98 | 56 |

^1^ "Randomised" was generated by distributing 1,592 regions of 734 bp randomly over the entire genome. This number and peak width mimics the number and average length of Rif1-∆C594 peaks in HU, where the largest number of the ChIP peaks were identified.

^2^ The number of Rif1 and Rif1-∆C594 peaks overlapping either S1-DRIP or predicted G4 sites was analysed, using BEDTools annotate. The number of "Randomised sites" overlapping S1-DRIP or predicted G4 sites was also analysed for comparison (last column).
