# Supplementary material for: Budding yeast Rif1 binds to replication origins and protects DNA at blocked replication forks
Source: EMBO Rep. 2018 Aug 13;19(9):e46222. doi: 10.15252/embr.201846222 (PMC6123642; doi:10.15252/embr.201846222)
Supplement: Supplementary file 4 — Table EV3 [file EMBR-19-e46222-s004.docx]

**Table EV3. Statistics of peak calling analysis**

|  | Number of peaks detected | |
| --- | --- | --- |
| Peak option | Narrow peak | Broad peak |
| Rif1; G1 | 762 | 798 |
| Rif1; HU | 915 | 853 |
| Rif1-∆C594; G1 | 883 | 958 |
| Rif1-∆C594; HU | 1592 | 1407 |
